# A Variable-Sampling Time Model Predictive Control Algorithm for Improving Path-Tracking Performance of a Vehicle

**DOI:** 10.3390/s21206845

**Published:** 2021-10-14

**Authors:** Yoonsuk Choi, Wonwoo Lee, Jeesu Kim, Jinwoo Yoo

**Affiliations:** 1 The Graduate School of Automotive Engineering, Kookmin University, Seoul 02707, Korea; dbstjr1020@kookmin.ac.kr (Y.C.); wonwoo@kookmin.ac.kr (W.L.); 2 Department of Congno-Mechatronics Engineering, Pusan National University, Busan 46241, Korea; Jeesukim@pusan.ac.kr; 3 Department of Optics and Mechatronics Engineering, Pusan National University, Busan 46241, Korea; 4 Department of Automobile and IT Convergence, Kookmin University, Seoul 02707, Korea

**Keywords:** model predictive control, variable sampling time, autonomous driving, path tracking, autonomous vehicle

## Abstract

This paper proposes a novel model predictive control (MPC) algorithm that increases the path tracking performance according to the control input. The proposed algorithm reduces the path tracking errors of MPC by updating the sampling time of the next step according to the control inputs (i.e., the lateral velocity and front steering angle) calculated in each step of the MPC algorithm. The scenarios of a mixture of straight and curved driving paths were constructed, and the optimal control input was calculated in each step. In the experiment, a scenario was created with the Automated Driving Toolbox of MATLAB, and the path-following performance characteristics and computation times of the existing and proposed MPC algorithms were verified and compared with simulations. The results prove that the proposed MPC algorithm has improved path-following performance compared to those of the existing MPC algorithm.

## 1. Introduction

Autonomously driving vehicles and electrification of vehicle parts have been hot topics in the automobile industry over the past few years, and many parts of vehicles have been replaced with electric devices. Accordingly, researchers are studying model predictive control (MPC) algorithms, which they apply to autonomous vehicles to track the vehicle’s driving route or optimize the efficiency of the engine, transmission, exhaust gas consumption, and motor performance. In the early 1960s, research on MPC involved the use of linear quadratic regulators designed by Kalman to minimize unconstrained quadratic objective functions. However, MPC was not applied in industry for a long time owing to the nonlinearity of actual industrial processes. Finally, in 1978 [1], chemical engineers applied MPC in chemical industrial control processes, thereby demonstrating its advantages over other control technologies.

When Ford Motor Company began exploring MPC-based control technology, MPC has already been applied in different automobile fields, such as for direct injection stratified engines [2] and traction control [3,4]. When user convenience became increasingly important and autonomously driving vehicles started to emerge, researchers studied optimal trajectories or collision avoidance trajectories by extending the use of MPC algorithms to the fields of advanced driver assistance systems (ADAS) [5,6,7] and autonomous driving [8,9]. These studies have demonstrated that constrained, multivariate MPC has advantages in following the path of autonomous vehicles [10].

MPC algorithms have been developed to improve the tracking performance by considering the nonlinearity of a vehicle model through nonlinear MPC or by further strengthening the state constraint such as with Robust MPC [11,12,13]. However, depending on the size of the prediction horizon, control horizon, and sampling time, the computation complexity can increase, or the tracking performance can be insufficient. In the above study, MPC was performed by fixing the size of the sampling time, and the size of the sampling time was set empirically. However, MPC using a fixed sampling time has improved tracking performance when the sampling time is small, but causes computational load. Conversely, when the sampling time is large, the computation time is fast, but the tracking performance is insufficient. The disadvantage of this method is that it is not suitable for vehicles that need to perform various actions in various scenarios.

As a result, subsequent research is using weight matrix, prediction horizon, and sampling time set as fixed parameters in MPC as variables, and changing and controlling prediction horizon or sampling time by applying arbitrarily designated prediction horizon and sampling time according to the conditions of each stage [14,15,16,17].

This paper proposes a variable sampling-time model predictive control algorithm (VST-MPC) for improving the path tracking performance of a vehicle, and the sampling time is adjusted based on the optimal steering angle and lateral acceleration inputs calculated with MPC. Previously published studies set the sampling time as constant or arbitrarily determined according to the conditions. However, the proposed VST-MPC algorithm adjusts the sampling time by using the gradient descent method according to the calculated optimal input. Therefore, it is controlled using various sampling times and designed to gradually converge to the minimum or maximum value. In addition, since the sampling time is changed based on the optimal input calculated from the MPC rather than the external parameters of the vehicle, it can be utilized in various environments and scenarios. This paper simulates four types of MPCs (MPC with a sampling time of 0.1, MPC with a sampling time of 0.2, MPC with a sampling time of 0.05, and the proposed MPC) in two scenarios. The simulation results show the path-following performance of the proposed MPC is improved compared to the MPC with a fixed sampling time. The algorithm was developed in the MATLAB environment, and the actual model utilized in the simulation used a vehicle among the car models provided by the Automated Driving Toolbox of MATLAB. The Automated Driving Toolbox of MATLAB provides many actors (cars, trucks, bicycles, and pedestrians). In addition, many of the vehicle parameters can be set, so various existing vehicles can be implemented by setting the parameters. Road design features and sensors can be used to collect driving data. The camera sensor was used to collect the reference data in this paper. The parameters of the vehicle and a more detailed description of the automated driving toolbox are given in Section 4.

The remainder of the paper is organized as follows. Section 2 discusses the basic MPC design to implement the proposed MPC. Section 3 describes the proposed VST-MPC algorithm. Section 4 describes the experimental environment and scenarios, and the results of each scenario. Finally, a conclusion is presented in Section 5.

## 2. Design of Model Predictive Control Algorithm

This section introduces the design of the MPC system. In Section 2.1, we present a bicycle model based on vehicle dynamics [18]. In Section 2.2, Section 2.3 and Section 2.4, the discrete linear vehicle model, cost function, and constraint for designing the MPC algorithm are presented.

### 2.1. Dynamic Bicycle Model of a Vehicle

**Figure 1:**
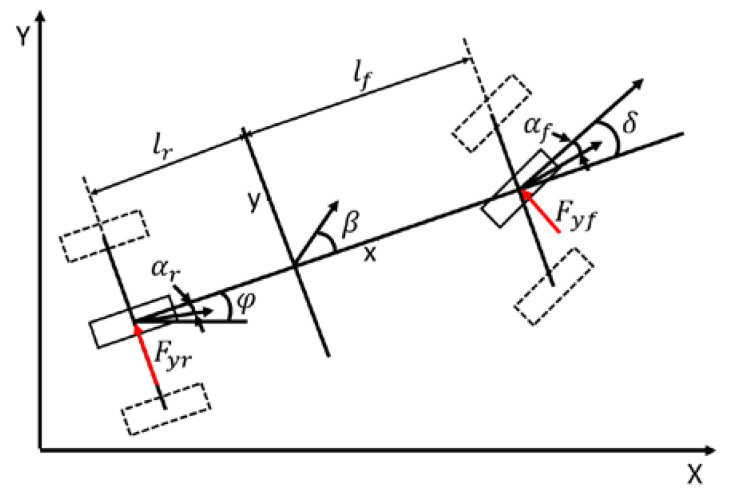
Bicycle model of a vehicle.

Figure 1 shows a bicycle model of a vehicle. The variables in the figure are:$\alpha_{f},{\;\alpha }_{r}$ are the tire slip angles of the front and rear wheels; β is the side-slip angle; δ is the steering angle of a vehicle; $F_{\mathrm{yf}},{\;F}_{\mathrm{yr}}$ are the front and rear tire forces; $l_{f},l_{r}$ are the longitudinal distances from the CG of vehicle to the front and rear wheels; and φ is the yaw angle.

This section presents the bicycle model of the vehicle used in the MPC design [19]. The following assumptions are made for the vehicle model [20]:

(1) The longitudinal velocity of the vehicle is constant.
(2) The left and right wheels (front and rear wheels) are considered single wheels.
(3) Suspension movement and slippage aerodynamic effects are approximately zero.
(4) The steering angle of the rear wheel is zero.

Based on these assumptions, a linear model of the vehicle can be obtained with Newton’s law (Figure 1)

(1) $$\;\dot{y}=\;\dot{y},$$

(2) $$\ddot{y}=-V_{x}\cdot \;\dot{\varphi }\;+a_{y},$$

(3) $$I_{z}\cdot \ddot{\varphi }=2\left(l_{f}\cdot F_{\mathrm{yf}}-l_{r}\cdot F_{\mathrm{yr}}\right),$$

where $V_{x}$ is the longitudinal velocity of a vehicle, $a_{y}$ is the lateral acceleration of the center of mass, $I_{z}$ is yaw moment of inertia, and $F_{\mathrm{yf}}$, $F_{\mathrm{yr}}$ are as follows:

(4) $$F_{\mathrm{yf}}=C_{\alpha f}\cdot \alpha_{f}=C_{\alpha f}\cdot \left(\delta -\frac{\dot{y}+l_{f}\cdot \dot{\varphi }}{V_{x}}\right),$$

(5) $$F_{\mathrm{yr}}=C_{\alpha r}\cdot \alpha_{r}=C_{\alpha r}\cdot \left(-\frac{\dot{y}-l_{r}\cdot \dot{\varphi }}{V_{x}}\right),$$

where $C_{\alpha f}$ is the cornering stiffness of front wheel, and $C_{\alpha r}$ is the cornering stiffness of rear wheel.

By substituting Equations (4) and (5) into Equation (3), the equation can be expressed as a vehicle model equation with the yaw rate ($\dot{\varphi }$) as shown below:

(6) $$\ddot{\varphi }=\frac{2\cdot l_{f}\cdot C_{\alpha f}-2\cdot l_{r}\cdot C_{\alpha r}}{I_{z}}\cdot \delta -\frac{2\cdot l_{f}\cdot C_{\alpha f}-2\cdot l_{r}\cdot C_{\alpha r}}{I_{z}\cdot V_{x}}\cdot \dot{y}-\frac{2\cdot l_{f}^{2}\cdot C_{\alpha f}+2\cdot l_{r}^{2}\cdot C_{\alpha r}}{I_{z}\cdot V_{x}}\cdot \dot{\varphi }.$$

Subsequently, the continuous state-space equation of a vehicle can be constructed. The MPC state-space equations are Equations (7) and (8):

(7) $$\dot{X_{C}}=A_{C}\cdot X_{C}+B_{C}\cdot \;u,$$

(8) $$\dot{Y_{C}}=C_{C}\cdot X_{C}+D_{C}\cdot \;u,$$

where

(9) $$X_{C}={\left[\begin{array}{cccc} y & \dot{y} & \varphi & \dot{\varphi } \end{array}\right]}^{T},$$

(10) $$A_{C}=\left[\begin{array}{cccc} 0 & 1 & 0 & 0\\ 0 & 0 & 0 & -V_{x}\\ 0 & 0 & 0 & 1\\ 0 & -\frac{2\cdot l_{f}\cdot C_{\alpha f}-2\cdot l_{r}\cdot C_{\alpha r}}{I_{z}\cdot V_{x}} & 0 & \frac{2\cdot l_{f}^{2}\cdot C_{\alpha f}+2\cdot l_{r}^{2}\cdot C_{\alpha r}}{I_{z}\cdot V_{x}} \end{array}\right],$$

(11) $$B_{C}=\left[\begin{array}{cc} 0 & 0\\ 1 & 0\\ 0 & 0\\ 0 & \frac{2\cdot l_{f}\cdot C_{\alpha f}}{I_{z}} \end{array}\right],{\;C}_{C}=\left[\begin{array}{cccc} 1 & 0 & 0 & 0\\ 0 & 1 & 0 & 0 \end{array}\right],$$

(12) $$\;u={\left[\begin{array}{cc} a_{y} & \delta \end{array}\right]}^{T},$$

where $X_{c}$ is the vehicle state; $A_{c}$ is the state-space matrix; $B_{c}$ and $C_{c}$ are the input and output matrices of the continuous-time state-space equation, respectively; and u is the vehicle’s control input. The vehicle states are described by the lateral position, lateral velocity, yaw angle, and yaw rate. The control inputs are the lateral acceleration of the center of mass and the front steering angle [21].

### 2.2. Discrete State-Space Vehicle Model for MPC

To convert the continuous-time state-space equation (introduced in Section 2.3) into a recursive equation, the value obtained by calculating the discrete-time state-space equation with the period $T_{s}$ is as follows [22]:

(13) $$X_{d}\left(k+1\right)=A_{d}\cdot X_{d}\left(k\right)+B_{d}\cdot \;u\left(k\right),$$

where

(14) $$A_{d}=e^{A_{d}\cdot T_{S}},{\;B}_{d}=\int_{0}^{T_{S}}e^{A_{C}\cdot \tau }\cdot B_{C}d\tau .$$

The output of the MPC algorithm is as follows:

(15) $$Y_{d}\left(k\right)=C_{d}\cdot X_{d}\left(k\right),$$

where

(16) $$Y_{d}\left(k\right)={\left[\begin{array}{cccc} y\left(k\right) & \dot{y}\left(k\right) & \varphi \left(k\right) & \dot{\varphi }\left(k\right) \end{array}\right]}^{T},$$

(17) $$C_{d}=C_{C}=\left[\begin{array}{cccc} 1 & 0 & 0 & 0\\ 0 & 1 & 0 & 0 \end{array}\right],$$

To improve the tracking performance in the path-following problem, Equations (13) and (15) are adjusted to the increment of the input:

(18) $$X_{a}\left(k+1\right)=A_{a}\cdot X_{a}\left(k\right)+B_{a}\cdot \Delta u\left(k\right),$$

(19) $$Y_{a}\left(k\right)=C_{a}\cdot X_{a}\left(k\right),$$

where

(20) $$X_{a}\left(k\right)=\left[\begin{array}{c} \Delta X_{d}\left(k\right)\\ Y_{d}\left(k\right) \end{array}\right],$$

(21) $$A_{a}=\left[\begin{array}{cc} A_{d} & O_{d}^{T}\\ C_{d}\cdot A_{d} & I_{d} \end{array}\right],{\;B}_{a}=\left[\begin{array}{c} B_{d}\\ C_{d}\cdot B_{d} \end{array}\right],{\;C}_{a}=\left[O_{d}I_{d}\right],$$

(22) $$O_{d}=\left[\begin{array}{cccc} 0 & 0 & 0 & 0\\ 0 & 0 & 0 & 0\\ 0 & 0 & 0 & 0\\ 0 & 0 & 0 & 0 \end{array}\right],{\;I}_{d}=\left[\begin{array}{cccc} 1 & 0 & 0 & 0\\ 0 & 1 & 0 & 0\\ 0 & 0 & 1 & 0\\ 0 & 0 & 0 & 1 \end{array}\right].$$

Equation (20) is a variable for the augmented state equation: $A_{a}$ is the augmented state matrix, $B_{a}$ is the augmented input matrix, and $C_{a}$ is the augmented output matrix; $O_{a}$ is a zero matrix, the number of rows of which is identical to the number of output variables and the number of columns is identical to the number of state variables. Finally, $I_{a}$ is the unit matrix, and its number of rows and columns correspond to the number of output variables.

### 2.3. Cost Function of MPC

To present the cost function in a simplified manner, the reference data and data predicted with the MPC algorithm are vectorized and expressed as follows:

(23) $$\Delta U_{m}={\left[\begin{array}{ccccc} \Delta u\left(k\right) & \ldots & \Delta u\left(k+m\right) & \ldots & \Delta u\left(k+N_{C}-1\right) \end{array}\right]}^{T},$$

(24) $$X_{m}\left(k\right)=X_{a}\left(k\right),$$

(25) $$Y_{m}\left(k\right)=F_{m}\cdot X_{m}\left(k\right)+G_{m}\cdot \Delta U_{m}\left(k\right)={\left[\begin{array}{ccc} Y_{a}\left(k+1\right) & \ldots & Y_{a}\left(k+N_{P}\right) \end{array}\right]}^{T},$$

where $N_{p}$ is the prediction horizon; $N_{c}$ is the control horizon; and $F_{m}$, $G_{m}$ are as below:

(26) $$F_{m}={\left[\begin{array}{cccccc} C_{a}A_{a} & C_{a}A_{a}^{2} & \ldots & C_{a}A_{a}^{N_{C}} & \ldots & C_{a}A_{a}^{N_{P}} \end{array}\right]}_{3\cdot N_{P}\times 10}^{T},$$

(27) $$G_{m}=\left[\begin{array}{cccc} C_{a}B_{a} & 0 & \cdots & 0\\ C_{a}A_{a}B_{a} & C_{a}B_{a} & \cdots & 0\\ \vdots & \vdots & \cdots & 0\\ C_{a}A_{a}^{N_{C}-1}B_{a} & C_{a}A_{a}^{N_{C}-2}B_{a} & \cdots & C_{a}B_{a}\\ \vdots & \vdots & \ddots & \vdots \\ C_{a}A_{a}^{N_{P}-1}B_{a} & C_{a}A_{a}^{N_{P}-2}B_{a} & \ldots & C_{a}A_{a}^{N_{P}-N_{C}}B_{a} \end{array}\right].$$

Subsequently, the MPC cost function is designed to find an input that minimizes the difference between the reference data and the state variable. The MPC cost function is as follows [23]:

(28) $$J={\left[R_{r}\left(k\right)-Y_{m}\left(k\right)\right]}^{T}\left[R_{r}\left(k\right)-Y_{m}\left(k\right)\right]+\Delta U_{m}^{T}\bar{R}\Delta U_{m},$$

where

(29) $$R_{r}\left(k\right)=\left[\begin{array}{c} y_{r}\left(k+1\right)\\ \dot{y_{r}}\left(k+1\right)\\ \varphi_{r}\left(k+1\right)\\ \dot{\varphi_{r}}\left(k+1\right)\\ \vdots \\ y_{r}\left(k+N_{P}\right)\\ \dot{y_{r}}\left(k+N_{P}\right)\\ \varphi_{r}\left(k+N_{P}\right)\\ \dot{\varphi_{r}}\left(k+N_{P}\right) \end{array}\right],{\;Y}_{m}\left(k\right)=\left[\begin{array}{c} y\left(k+1\right)\\ \dot{y}\left(k+1\right)\\ \varphi \left(k+1\right)\\ \dot{\varphi }\left(k+1\right)\\ \vdots \\ y\left(k+N_{P}\right)\\ \dot{y}\left(k+N_{P}\right)\\ \varphi \left(k+N_{P}\right)\\ \dot{\varphi }\left(k+N_{P}\right) \end{array}\right].$$

To minimize the cost function, its derivative must be zero:

(30) $$\frac{\partial J}{\partial X_{m}}=0,\frac{\partial J}{\partial \Delta U_{m}}=0.$$

By substituting Equation (30) into Equation (28), the cost function for $\Delta U_{m}$ can be obtained as

(31) $$J=\frac{1}{2}\Delta U_{m}^{T}E_{m}\Delta U_{m}+\Delta U_{m}^{T}K_{m},$$

where

(32) $$E_{m}=2\left(G_{m}^{T}G_{m}+\;\bar{R}\right),$$

(33) $$K_{m}=-2\cdot G_{m}^{T}\cdot \left[R_{r}\left(k\right)-F_{m}\cdot X_{m}\left(k\right)\right].$$

### 2.4. Constraints of the MPC Algorithm

The MPC algorithm calculates the optimized control input according to a given cost function and the specific state variables and constraints. Thus, it sets constraints on the state variables and inputs [24]. Because the cost function is modified in terms of ΔU_m_, the constraint must be expressed in terms of ΔU_m_. The constraint of the control input vector is as follows:

(34) $$U_{\min}\leq U_{m}\leq U_{\max},$$

(35) $$-U_{m}\leq -U_{\min},{\;U}_{m}\leq U_{\max}.$$

Equation (34) includes two inequality constraints, and it is identical to Equation (35). Furthermore, Equation (35) can be expressed as a matrix:

(36) $$\left[\begin{array}{c} -I\\ I \end{array}\right]U_{m}\leq \left[\begin{array}{c} -U_{\min}\\ U_{\max} \end{array}\right].$$

$U_{m}$ can be expressed as follows:

(37) $$\left[\begin{array}{c} u\left(k\right)\\ u\left(k+1\right)\\ u\left(k+2\right)\\ \vdots \\ u\left(k+N_{C}-1\right) \end{array}\right]=\left[\begin{array}{c} I\\ I\\ I\\ \vdots \\ I \end{array}\right]u\left(k-1\right)+\left[\begin{array}{ccccc} I & 0 & 0 & \cdots & 0\\ I & I & 0 & \cdots & 0\\ I & I & I & \cdots & 0\\ \vdots & \vdots & \vdots & \ddots & \vdots \\ I & I & I & \cdots & I \end{array}\right]\left[\begin{array}{c} \Delta u\left(k\right)\\ \Delta u\left(k+1\right)\\ \Delta u\left(k+2\right)\\ \vdots \\ \Delta u\left(k+N_{C}-1\right) \end{array}\right].$$

By substituting Equation (37) into Equation (36), the following constraints can be obtained as

(38) $$\left[\begin{array}{c} -C_{2}\\ C_{2} \end{array}\right]\Delta U_{m}\leq \left[\begin{array}{c} -U_{\min}+C_{1}u\left(k-1\right)\\ U_{\max}-C_{1}u\left(k-1\right) \end{array}\right],$$

where

(39) $$C_{1}={\left[\begin{array}{c} 1\\ 1\\ 1\\ \vdots \\ 1 \end{array}\right]}_{N_{C}\times 3},{\;C}_{2}={\left[\begin{array}{ccccc} I & 0 & 0 & \cdots & 0\\ I & I & 0 & \cdots & 0\\ I & I & I & \cdots & 0\\ \vdots & \vdots & \vdots & \ddots & \vdots \\ I & I & I & \cdots & I \end{array}\right]}_{N_{C}\times N_{C}}.$$

By following the same process for the increment of the input and output, the following inequality constraint can be obtained as

(40) $$\left[\begin{array}{c} -I\\ I \end{array}\right]\cdot \Delta U_{m}\leq \left[\begin{array}{c} -\Delta U_{\min}\\ \Delta U_{\max} \end{array}\right],$$

(41) $$\left[\begin{array}{c} -G_{m}\\ G_{m} \end{array}\right]\Delta U_{m}\leq \left[\begin{array}{c} -Y_{\min}+F_{m}X_{m}\left(k\right)\\ Y_{\max}-F_{m}X_{m}\left(k\right) \end{array}\right].$$

By using Equations (38), (40) and (41), the MPC constraints can be derived:

(42) $$\left[\begin{array}{c} M_{1}\\ M_{2}\\ M_{3} \end{array}\right]\Delta U_{m}\leq \left[\begin{array}{c} N_{1}\\ N_{2}\\ N_{3} \end{array}\right],$$

where

(43) $$M_{1}=\left[\begin{array}{c} -C_{2}\\ C_{2} \end{array}\right],{\;M}_{2}=\left[\begin{array}{c} -I\\ I \end{array}\right],{\;M}_{3}=\left[\begin{array}{c} -G_{m}\\ G_{m} \end{array}\right],$$

(44) $$N_{1}=\left[\begin{array}{c} -U_{\min}+C_{1}u\left(k-1\right)\\ U_{\max}-C_{1}u\left(k-1\right) \end{array}\right],$$

(45) $$N_{2}=\left[\begin{array}{c} -\Delta U_{\min}\\ \Delta U_{\max} \end{array}\right],$$

(46) $$N_{3}=\left[\begin{array}{c} -Y_{\min}+F_{m}X_{m}\left(k\right)\\ Y_{\max}-F_{m}X_{m}\left(k\right) \end{array}\right].$$

## 3. A Variable Sampling-Time Model Predictive Control (VST-MPC) Algorithm

In MPC, the sampling time determines how many times the MPC runs the control algorithm during a driving scenario. When the sampling time is short, because the control algorithm is run by dividing the scenario into a long sequence, it can respond quickly to disturbances and improve the tracking performance. However, the computation time is long. When the sampling time is long, and the computation time is shortened because the scenario is divided into a short sequence; however, the tracking performance is insufficient, and the algorithm cannot respond quickly to disturbances [25]. In this section, we propose the VST-MPC method, which adjusts the sampling time based on the optimal input value calculated by the MPC algorithm.

### 3.1. Proposed VST-MPC Algorithm Using Optimal Input Sequence

The VST-MPC algorithm is designed to run while adjusting the sampling time according to the driving situation at each stage. The basic VST-MPC algorithm is as follows: In a straight driving section, because the input steering angle and lateral acceleration are approximately zero, the sampling time does not have much effect on the tracking performance and influence of the disturbance. Thus, the sampling time is set to the maximum in the straight driving section for faster computation. In the curved driving section, because the input steering angle and lateral acceleration change continuously, the sampling time affects the tracking performance and reactivity toward the disturbance. Therefore, the sampling time is gradually decreased to improve the tracking performance in the curved driving section.

Algorithm 1 presents the pseudo-code of the VST-MPC algorithm. The description of the pseudo-code is as follows: In Step 1, the default sampling time is set to 0.2. In Step 2, the parameters of the MPC algorithm (various parameters such as $A_{d}$, $B_{d},{\;C}_{d},{\;D}_{d},{\;F}_{m},{\;G}_{m}$) are set, and the MPC algorithm computes the optimal input to predict the next move. In Step 3, the control input calculated with the MPC algorithm is used to predict the next move of the vehicle. In Step 4, the predicted state is stored as the past state, and the control input is stored as the past input. In Step 5, the sampling time to be used when the next vehicle move is predicted is calculated with the calculated inputs $u_{1}=a_{y}=$ $\frac{\Delta V_{y}}{T_{s}}$ and $u_{2}=\delta$. The contents of the equations of the fifth step are described in detail in Section 3.2. In Step 6, the sampling time calculated in Step 5 is set as the sampling time for the next prediction of the MPC. Step 7 prevents the sampling time from continuously increasing or decreasing. When the sampling time calculated in Step 5 exceeds the maximum value of the sampling time, the sampling time for the next prediction of the MPC is set as the maximum value of the sampling time. In contrast, when the sampling time calculated in Step 5 exceeds the minimum value of the sampling time, the sampling time for the next prediction of the MPC is set as the minimum value of the sampling time. In Step 7, the algorithm returns to Step 2 and continues to predict the next move of the vehicle from Step 2 to Step 7 until the scenario is over.
**Algorithm 1** The Pseudo Code of VST-MPC.Step 1: Set the initial sampling time $T_{S}=0.2$Step 2: Set the MPC parameter and calculate the optimal input with MPCStep 3: Predict $X_{a}\left(K+1\right)=A_{a}\cdot X_{a}\left(K\right)+B_{a}\cdot u\left(K\right)$Step 4: Update X_past_ = X_a_ (K + 1), u_past_ = u(K)Step 5: Calculate the next sampling time using the following equation.$T_{S}\left(K+1\right)=T_{S}\left(K\right)+\mathrm{sign}\left(Z-C\right)\cdot \min\left(Z,\;C\right)$where $Z=-\lambda \cdot \left|\frac{\partial }{\partial T_{S}}\left(\delta \left(K\right)\cdot \frac{\cdot V_{y}\left(K\right)}{T_{S}\left(K\right)}\right)\right|,\;C=-0.01$Step 6: Set $T_{S}$ = $T_{S}\left(K+1\right)$Step 7: If     T_s_ > $T_{S,\mathrm{maximum}}$ $T_{S}$ = $T_{S,\mathrm{maximum}}$
else if   $T_{S}$ < $T_{S,\mathrm{minimum}}$ $T_{S}$ = $T_{S,\mathrm{minimum}}$
endStep 8: Go to Step 2 until MPC iteration is over.

### 3.2. Design a Function of VST-MPC for Sampling Time Variation

This section presents the equation for setting the sampling time in the VST-MPC algorithm:

(47) $$T_{S}\left(K+1\right)=T_{S}\left(K\right)+\mathrm{sign}\left(Z-C\right)\cdot \min\left(Z,\;C\right),$$

where

(48) $$\frac{\Delta V_{y}}{\Delta T_{s}}=u_{1}=a_{y},\;\delta =u_{2},$$

(49) $$Z=-\lambda \cdot \left|\frac{\partial }{\partial T_{S}}\left(\delta \left(K\right)\cdot \frac{\Delta V_{y}\left(K\right)}{T_{S}\left(K\right)}\right)\right|,$$

(50) $$\mathrm{sign}\left(X\right)≜\{\begin{array}{cc} 1 & \left(X>0\right)\\ 0 & \left(X=0\right)\\ -1 & \left(X<0\right) \end{array},$$

where λ and C are manually determined constants. The values of λ and C are described in Section 4.1.

In Equation (48), $u_{1}$ and $u_{2}$ are the inputs calculated by solving the cost function. This expression is designed to change the sampling time according to the control input. In the driving scenario, the inputs $a_{y}$ and δ converge to 0 in the straight driving section. However, in the curved driving section, the inputs $a_{y}$ and δ have non-zero values. Equation (47) reduces the computation time by maintaining a long sampling time in the straight driving section owing to these driving characteristics. In the curved driving section, the sampling time is gradually decreased according to the calculated input to improve the tracking performance and for reliable reactivity to sudden disturbances.

In other words, in a driving scenario starting when the sampling time is set to 0.2, Z is zero because the control input is close to zero in the straight driving scenario. The next sampling time is predicted by adding the selected C value to the existing sampling time of 0.2. However, 0.2 is maintained owing to the maximum value condition of the sampling time in Step 7 of the pseudo-code. In the curved driving scenario, the control input gradually increases. Thus, the new sampling time is determined by subtracting Z from the equation from the existing sampling time 0.2.

In general, the path tracking error of MPC increases over curved sections. Therefore, to reduce the path-tracking error, it is necessary to significantly reduce the sampling time when the input calculated through MPC is large. In other words, the sampling time should change significantly in the curved driving section where the calculated inputs are increasing. However, when the sampling time is changed rapidly, the inputs will also change rapidly, reducing the ride quality. There may be situations where the MPC cannot compute the inputs. To prevents this phenomenon, the sampling time should be changed by a small increment at the first changing point from the linear driving section to the curved driving section, and the sampling time should be gradually changed according to the input. For this reason, in the first point where the sampling time changes, λ is set so that the sampling time, which is the smallest possible increment, changes to 0.01. For the same reason, C is set as the smallest increment so that the sampling time is adjusted step by step. The value of C is set to 0.01 when the input is 0, that is, to increase the sampling time in the straight section. Therefore, C is set to −0.01 to add to the existing sampling time.

Equation (50) determines the sign of the calculated expression. The reason for selecting the smaller of the values C and Z in Equation (47) is to cause the sampling time that converges to 0.05 in a curved driving section to converge to 0.2 again in a straight driving section. Therefore, when C is selected, the calculated expression must be added to the existing sampling time. Conversely, when C is not selected, it is set to subtract the calculated equation from the existing sampling time, as it indicates a curved driving section. The gradient descent algorithm is used in this equation such that the sampling time gradually converges to the minimum bound, 0.05, or maximum bound, 0.2, as the curvature of the driving path changes [26]. In the curved driving section, the sampling time decreases to 0.05. In the straight driving section, the sampling time increases to 0.2.

## 4. Configuration Driving Scenario Using MATLAB and Simulations

In this section, the proposed VST-MPC algorithm presented in Section 2 and Section 3 is verified based on different scenarios. Section 4.1 describes the scenario, and Section 4.2 presents the experimental results.

The simulation was performed with the following environment:

- CPU: AMD Ryzen 7 3800XT 8-Core Processor 3.90 GHz
- RAM: 32.0 GB
- GPU: NVIDIA GeForce RTX 3070
- RAM: 32.0 GB
- Tool: MATLAB 2020b, Automated Driving Toolbox

For the scenario configuration and simulations, MATLAB and Automated Driving Toolbox be linked. The Automated Driving Toolbox of MATLAB provides many actors (car, truck, bicycle, and pedestrian). In this paper, the car was used for the vehicle model for simulations. The camera sensor was used for collecting the reference data of the simulations. The Automated Driving Toolbox can be used to set many vehicle parameters and can collect the driving data through road design and sensors. The toolbox provides several algorithms and functions that facilitate the simulation of ADAS and autonomous driving systems (static and dynamic actors, various sensors, etc.). In addition, some visualization environments such as 3D simulation and bird’s eye view are provided. Scenarios can also be exported to MATLAB in the form of functions, making it easy to link with MATLAB and to plot experimental results on MATLAB. Because of these advantages, it is used in ADAS and autonomous driving system verification in various papers [27].

Table 1 shows the parameters of a vehicle model and input constraints. Because the scenario was performed at a speed of 20 m/s, the maximum acceleration constraint was set using 2.24 m/s^2^, the empirical maximum acceleration value of a petrol car driving at 20 m/s. The minimum acceleration constraint was set using 3.97 m/s^2^, the empirical maximum deceleration value of a petrol car driving at 20 m/s [28]. The maximum steering angle constraint was set by using Ackerman Jeantaud geometry in that the minimum radius of gyration is set to 6 m [29].

**Figure 2:**
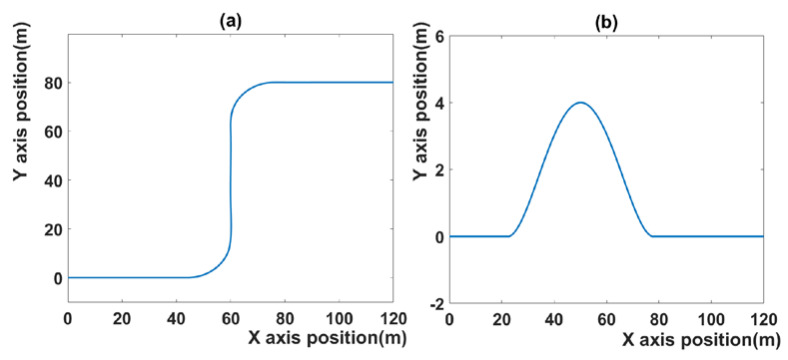
(**a**) Trajectory of scenario 1, (**b**) trajectory of scenario 2.

Figure 2 shows the trajectory of scenario 1 and scenario 2. A detailed description of the scenario is provided in Section 4.1. Most of the MPC-related papers use the sampling time as 0.1 [30]. Therefore, 0.1 is also used in this paper. Additionally, the sampling time, 0.2 was selected for comparison between the proposed MPC and the MPC with a longer sampling time than 0.1. The sampling time, 0.2 is used at tracking control system and cruise control system [31,32].

### 4.1. Scenario Description

In this section, the results of the MPC system with 0.2, 0.1, and 0.05 sampling times and those of the proposed VST-MPC system for each scenario are compared.

The first scenario is shown in Figure 2a: the vehicle runs on two curved roads in a row. In the beginning, the vehicle runs on a straight road of 40 m length and turns left into a curved driving section with a 20 m radius of gyration. Subsequently, it runs on a 40 m straight driving section and enters a (right-turning) curved section with a 20 m radius of gyration. The scenario ends when the 40 m straight driving section has been passed. The initial speed of the vehicle is 20 m/s, the prediction horizon is 10, and the control horizon is 2. The prediction horizon indicates the amount of data that MPC references to calculate the next prediction. The prediction horizon affects tracking performance and computation time. However, in this paper, it is set as a fixed constant. Many papers set the prediction horizon to 10 [7,20]. In an MPC system with a prediction horizon equal to 10, tracking performance and computation time are reasonable, so the prediction horizon is set to 10. The value of λ, which is a constant in Equation (47) is 0.0045, and C, which is a constant in Equation (47), is −0.001. In addition, the experiment is performed at different sampling times: 0.2, 0.1, and a variable sampling time.

The second scenario is shown in Figure 2b. This scenario consists of two driving lanes. At the start of the scenario, the vehicle runs on the second lane of a two-lane road. It switches to the first lane after detecting an obstacle at approximately 50 m distance from the start and returns to the second lane after passing the obstacle. The initial speed of the vehicle is 20 m/s, the prediction horizon is 10, and the control horizon is 2. In the second scenario, most of the parameters are the same except that λ is 0.02, which is set empirically. The results of the four MPC algorithms with different sampling times are compared to assess the tracking performance of each algorithm.

### 4.2. Simulation Results of Scenario 1

**Figure 3:**
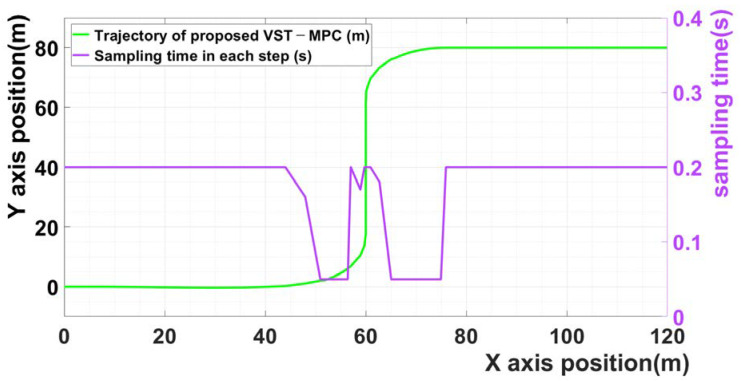
The sampling time of scenario 1 in each step.

Figure 3 shows the sampling time of scenario 1 in each step. The violet curve is the sampling time in each step. The results indicate that the sampling time changes based on the calculated input values of the lateral acceleration and steering angle when the vehicle enters the curved driving section. Owing to the effect of the proposed MPC, a low sampling time was selected for the curved driving section, and a high sampling time was selected for the straight driving section.

**Figure 4:**
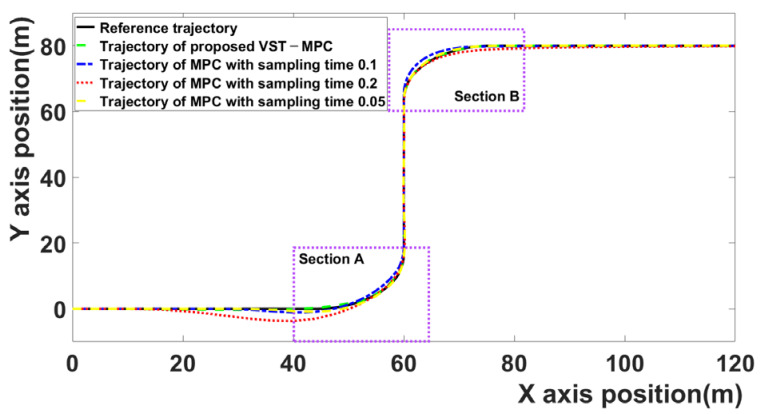
Comparison of trajectories of four MPC algorithms.

Figure 4 compares the trajectories of the four MPC algorithms. The black curve is the reference trajectory, the red curve represents the MPC algorithm with 0.2 sampling time, the blue curve represents the MPC algorithm with 0.1 sampling time, the green curve represents the results of the proposed MPC algorithm, and the brown curve represents the result of the MPC algorithm with 0.05 sampling time. Because discussing the tracking error of each MPC algorithm based on a figure that shows the entire trajectories is difficult, the tracking errors of each section are discussed separately.

**Figure 5:**
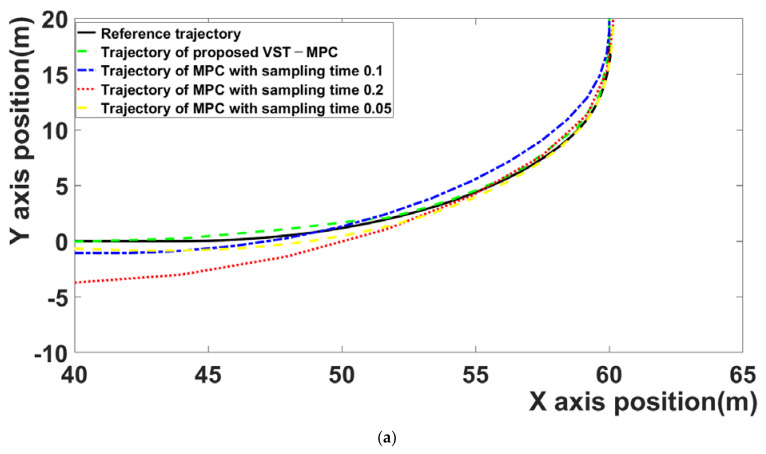

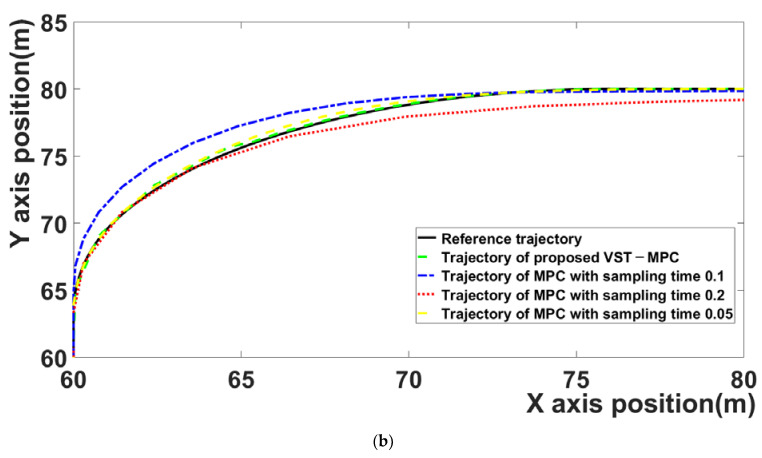
(**a**) The trajectory of section A, (**b**) the trajectory of section B.

Figure 5a is section A. Section A is the section entering from the first straight driving area to the first curved driving area (35–65 m on the *X* axis).

Figure 5b is section B. Section B is the section entering from the second straight driving area to the second curved driving area (50–80 m on the *X* axis).

The proposed VST-MPC is closest to the reference trajectory compared to the MPC with a 0.1 sampling time and the MPC with a 0.2 sampling time. This indicates that the path-following performance is improved by the effect of the proposed VST-MPC. The proposed VST-MPC shows similar tracking performance to the MPC with a sampling time of 0.05. However, in terms of computation time, the proposed MPC improves over the MPC with a sampling time of 0.05. This is discussed in detail in Table 2.

**Figure 6:**
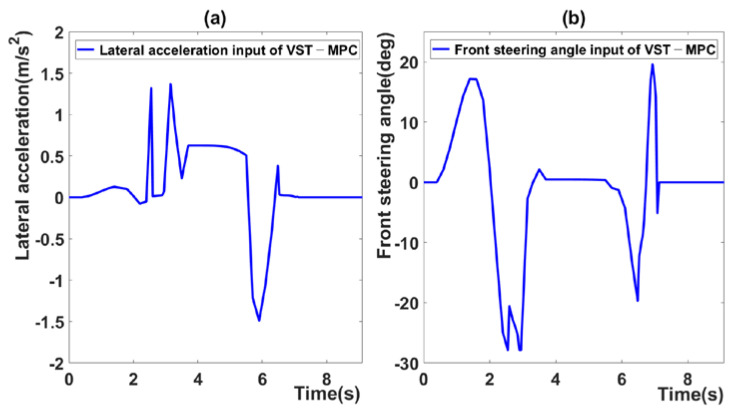
(**a**) Lateral acceleration input of VST-MPC, (**b**) steering angle input of VST-MPC.

Figure 6 presents the control inputs of the proposed MPC. The value tends to change rapidly in the section in which the sampling time is changed. That is, when the sampling time is long, the control input is large because the reference for predicting the next step changes to a large increment. When the sampling time is short, the calculated control input is small because the reference for predicting the next step relatively changes to a small increment. In the proposed MPC algorithm, because the sampling time changes continuously, the control input increases or decreases according to the sampling time. Thus, the input value tends to change rapidly.

Although this does not pose a major safety risk, this problem must be addressed in further studies to ensure passenger comfort.

**Figure 7:**
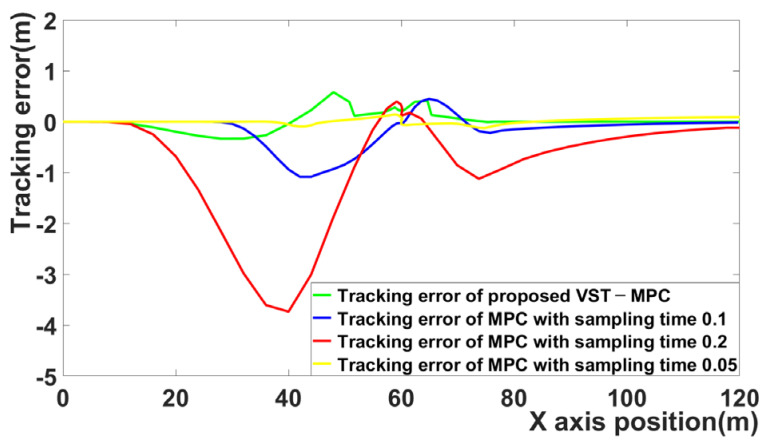
Comparison of tracking error for four MPC algorithms.

Figure 7 is the tracking error for the four MPCs. The tracking error of MPC with a 0.1 sampling time and MPC with a 0.2 sampling time increases in the curved driving section. Figure 7 shows that the tracking errors of VST-MPC and MPC with sampling time 0.05 are the smallest.

The average tracking error and computation time are determined when the MPC algorithm has completed all cycles. The average tracking error is calculated using the average absolute error.

Table 2 shows the average tracking errors and computation times of the four MPC algorithms. The sampling time affects the average tracking error and computation time. When the sampling time is long, there is an advantage in that the computation time is decreased. However, there is a disadvantage in that the tracking error is increased. In the opposite case, when the sampling time is short, there is an advantage in that the tracking error is decreased. However, there is a disadvantage in that the computation time is increased.

The MPC algorithm with a 0.2 sampling time has the disadvantage of a fairly large average tracking error, 0.6407 m, but it has the advantage of a short computation time, 0.1292 s.

The MPC algorithm with a 0.1 sampling time shows adequate performance with an average tracking error of 0.1617 m. In addition, the computation time is 0.2880 s, maintaining an appropriate level.

The MPC algorithm with a 0.05 sampling time has the advantage of the smallest average tracking error, 0.1344 m, but it has the disadvantage of a long computation time, 0.4170 s.

The computation time of the proposed MPC algorithm is increased compared to the computation time of the MPC algorithm with a 0.2 sampling time and the MPC algorithm with a 0.1 sampling time. However, the average tracking performance of the proposed MPC algorithm is improved compared to the average tracking performance of the MPC algorithm with 0.2 sampling time and the MPC algorithm with 0.1 sampling time. The computation time of the proposed MPC algorithm is decreased compared to the computation time of the MPC algorithm with a sampling time of 0.05. The average tracking performance of the proposed MPC algorithm is similar to that of the MPC algorithm with a 0.05 sampling time. This confirms that the computation time can be shortened while maintaining similar performance in terms of tracking performance when the proposed VST-MPC algorithm and the MPC algorithm with the shortest sampling time are compared.

The path-tracking error of the proposed VST-MPC was reduced by about 14% compared to the MPC with a 0.1 sampling time and was reduced by about 351% compared to the MPC with a 0.2 sampling time. However, the path-tracking error of the proposed VST-MPC was increased only by about 5% compared to the MPC with a 0.05 sampling time. The computation time was increased by about 12% compared to the MPC with a 0.1 sampling time and was increased by about 153% compared to the MPC with a 0.2 sampling time. However, the computation time was reduced by about 28% compared to the MPC with a 0.05 sampling time. Due to the effect of the proposed VST-MPC algorithm, the tracking performance is increased compared to the MPC with a 0.1 sampling time and MPC with a 0.2 sampling time. And it shows performance similar to the tracking performance of MPC with a sampling time of 0.05. The proposed VST-MPC algorithm has a disadvantage in that it increases the computation time, but this is low compared to the increase in tracking performance. The proposed VST-MPC algorithm can reduce the computation time compared to MPC with a 0.05 sampling time. That is, the code that changes the sampling time in the proposed MPC algorithm does not significantly affect the computation time, and only the changed sampling time increases the computation time. This means that the proposed VST-MPC has the advantages of both MPC with long sampling time and MPC with short sampling time.

### 4.3. Simulation Result of Scenario 2

**Figure 8:**
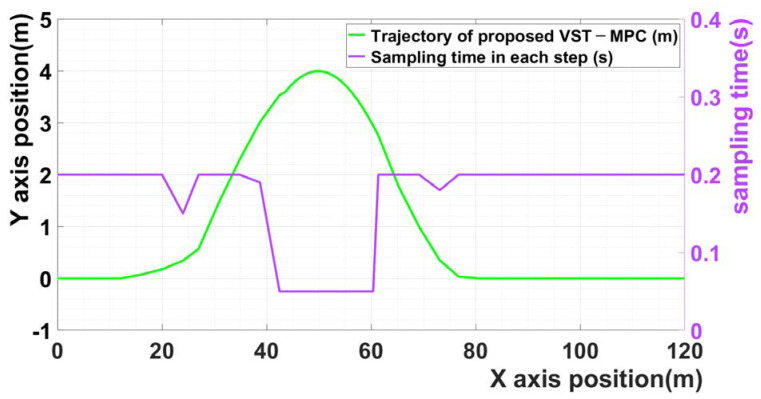
The sampling time of scenario 2 in each step.

Figure 8 shows the sampling times for Scenario 2 in each stage. The violet curve is the sampling time in each step. The results indicate that the sampling time changes according to the calculated lateral acceleration and steering angle input values when the vehicle enters a curved driving section. Due to the effect of the proposed MPC, a low sampling time was selected for the curve driving section and a high sampling time was selected for the straight driving section. In addition, in the section that is the inflection point in the trajectory, the sampling time is increased again because the steering angle and the lateral acceleration are close to zero.

**Figure 9:**
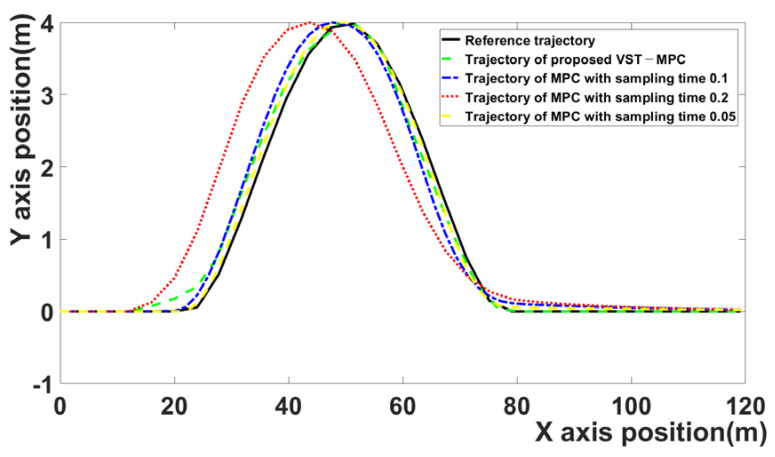
Comparison of trajectories of four MPC algorithms.

Figure 9 compares the trajectories of the four MPC algorithms. The black curve is the reference trajectory, the red curve represents the MPC algorithm with a 0.2 sampling time, the blue curve represents the MPC algorithm with a 0.1 sampling time, the green curve represents the proposed MPC algorithm, and the brown curve represents the MPC algorithm with a 0.05 sampling time. The proposed VST-MPC is closest to the reference trajectory compared to the MPC with a 0.1 sampling time and the MPC with a 0.2 sampling time. This indicates that the path-following performance is improved by the effect of the proposed VST-MPC. Additionally, the proposed VST-MPC shows similar tracking performance to the MPC with a sampling time of 0.05. However, in terms of computation time, the proposed MPC improves over the MPC with a sampling time of 0.05. This is discussed in detail in Table 3.

**Figure 10:**
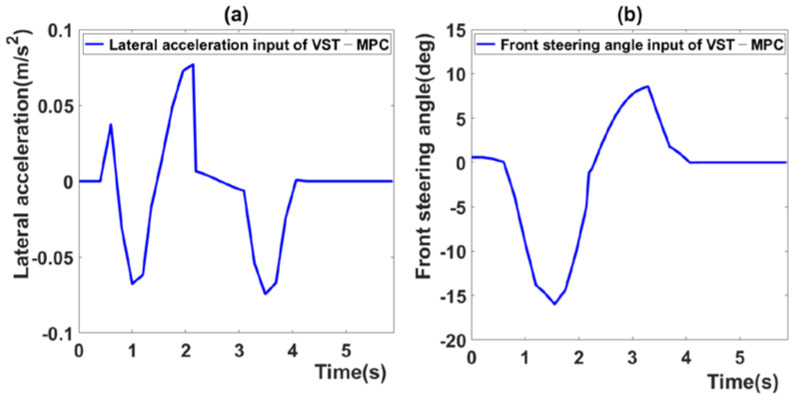
(**a**) Lateral acceleration input of VST-MPC in scenario 2, (**b**) steering angle input of VST-MPC in scenario 2.

Figure 10 presents the control input of Scenario 2. As noted in Section 4.2, the control input changes suddenly, according to the sampling time.

**Figure 11:**
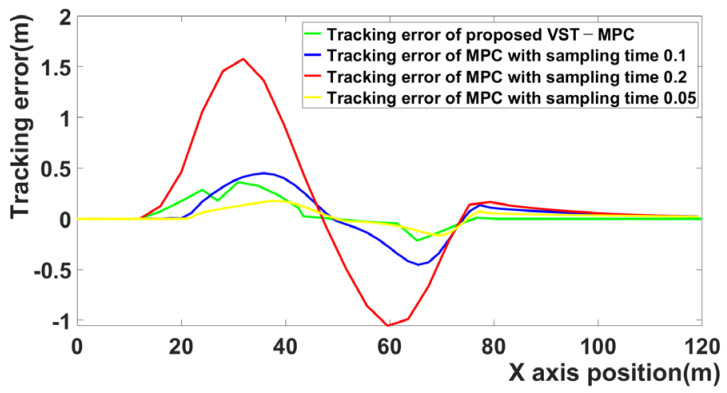
Comparison for tracking error of four MPC algorithms.

Figure 11 shows the tracking errors of the four MPC algorithms. When the sampling time is 0.2 and 0.05, the tracking error is large. By contrast, the proposed VST-MPC algorithm does not have a large tracking error. Even compared to MPC with a 0.05 sampling time, the tracking error of the proposed VST-MPC is similar with the MPC with a 0.05 sampling time.

The average tracking error and computation time are measured when the MPC has completed all cycles.

Table 3 shows the average tracking errors and computation times of the four MPC algorithms. The sampling time affects the average tracking error and computation time.

The MPC algorithm with 0.2 sampling time has the disadvantage of a fairly large average tracking error, 0.4079 m, but it has the advantage of a short computation time, 0.0883 s.

The MPC algorithm with a 0.05 sampling time has the advantage of the smallest average tracking error, 0.0556 m, but it has the disadvantage of a long computation time, 0.3121 s.

The proposed MPC algorithm has improved tracking performance over the MPC algorithm with 0.1 sampling time and the MPC algorithm with 0.2 sampling time.

The computation time of the proposed MPC algorithm is decreased compared to the computation time of the MPC algorithm with a sampling time of 0.05. The average tracking performance of the proposed MPC algorithm is similar to that of the MPC algorithm with a 0.05 sampling time. This confirms that the computation time can be shortened while maintaining similar performance in terms of tracking performance when the proposed VST-MPC algorithm and the MPC algorithm with the shortest sampling time are compared.

The tracking error of the proposed VST-MPC was reduced by about 145% compared to the MPC with a 0.1 sampling time and 616% compared to the MPC with a 0.2 sampling time. However, the path-tracking error of the proposed VST-MPC was increased only by about 2% compared to the MPC with a 0.05 sampling time. The computation time was increased by about 42% compared to MPC with a 0.1 sampling time and by 138% compared to MPC with a 0.2 sampling time. However, the computation time was reduced by about 54% compared to the MPC with a 0.05 sampling time. Due to the effect of the proposed VST-MPC algorithm, the tracking performance is improved over that of MPC with a 0.1 sampling time and MPC with a 0.2 sampling time. And it shows performance similar to the tracking performance of MPC with a sampling time of 0.05. The proposed VST-MPC algorithm has a disadvantage in that it increases the computation time, but this is low compared to the increase in tracking performance. The proposed VST-MPC algorithm can reduce computation time compared to MPC with a 0.05 sampling time. That is, the code that changes the sampling time in the proposed MPC algorithm does not significantly affect the computation time, and only the changed sampling time increases the computation time. This means that the proposed VST-MPC has the advantages of both MPC with long sampling time and MPC with short sampling time.

## 5. Conclusions and Future Work

This paper proposed an MPC algorithm for autonomous vehicles. The algorithm adjusts the sampling time based on the lateral acceleration and steering angle, which are the inputs calculated by the MPC algorithm when the vehicle is running. When a short sampling time is chosen, the algorithm can cope well with sudden disturbances and improve its tracking performance, however, this prolongs the computation time. When the sampling time is long, the computation time is shortened. However, the algorithm cannot cope well with sudden disturbances and exhibits poor tracking performance. To compensate for these deficiencies, this paper proposes the VST-MPC algorithm. This algorithm adjusts the sampling time of each step using optimized input calculated by the MPC algorithm when driving the vehicle. The tracking characteristics and computation time of the proposed and conventional MPC algorithms were compared with two fixed sampling times in two scenarios. According to the results, the proposed MPC algorithm shows improving tracking performance and similar computation time to MPC with a sampling time of 0.1. This means that the proposed VST-MPC has both advantages of MPC with short sampling time and MPC with long sampling time.

Because the vehicle is driven by continuously changing the sampling time, the input lateral acceleration and the front wheel steering angle are continuously changed. Although this does not affect the safety of the passengers, it may diminish driving comfort. Therefore, the research to improve this is being planned. In addition, the research using HiLS or real-time simulation is being planned for better proof of the algorithm.

## Figures and Tables

**Table 1:** MPC parameters.

Symbol	Description	Value [units]
m	Vehicle mass	2020 [kg]
l_f_	C.g. distance to front wheel	1.40 [m]
l_r_	C.g. distance to rear wheel	1.65 [m]
I_z_	Yaw moment of inertia	3234 [kg · m^2^]
C_αf_	Front wheel cornering stiffness	1420$\cdot \frac{180}{\pi }$ [N]
C_αr_	Rear wheel cornering stiffness	1420$\cdot \frac{180}{\pi }$ [N]
V	Velocity of vehicle	20 [m/s]
a_y,max_	Maximum acceleration constraint	2.24 [m/s^2^]
a_y,min_	Minimum acceleration constraint	−3.97 [m/s^2^]
δ_f,max_	Maximum steering angle constraint	0.4864 [rad]
δ_f,min_	Minimum steering angle constraint	−0.4864 [rad]

**Table 2:** Average tracking error and entire computation time of four MPC algorithms.

The MPC Algorithm	Average Tracking Error (m)	Computation Time (s)
The MPC algorithm with sampling time 0.1	0.1617	0.2880
The MPC algorithm with sampling time 0.2	0.6407	0.1292
The MPC algorithm with sampling time 0.05	0.1344	0.4170
VST-MPC	0.1420	0.3267

**Table 3:** Average tracking error and entire computation time of four MPC algorithms.

The MPC Algorithm	Average Tracking Error (m)	Computation Time (s)
The MPC algorithm with sampling time 0.1	0.1395	0.1430
The MPC algorithm with sampling time 0.2	0.4079	0.0883
The MPC algorithm with sampling time 0.05	0.0556	0.3121
VST-MPC	0.0570	0.2033

## Data Availability

Not applicable.
